# Three-dimensional reconstruction of electron micrographs reveals intrabulbar circuit differences between accessory and main olfactory bulbs

**DOI:** 10.3389/fnana.2013.00005

**Published:** 2013-04-22

**Authors:** Keiko Moriya-Ito, Kentaroh Endoh, Yoko Fujiwara-Tsukamoto, Masumi Ichikawa

**Affiliations:** ^1^Department of Dementia and Higher Brain Function, Tokyo Metropolitan Institute of Medical ScienceSetagaya, Tokyo, Japan; ^2^Center of Basic Technology Research, Tokyo Metropolitan Institute of Medical ScienceSetagaya, Tokyo, Japan; ^3^Basic Brain Science Research Center, Brain Science Institute, Tamagawa UniversityMachida, Tokyo, Japan; ^4^Laboratory of Neural Circuitry, Graduate School of Brain Science, Doshisha UniversityKizugawa, Kyoto, Japan

**Keywords:** dendrodendritic reciprocal synapses, dendritic spines, gemmules, self-inhibition, electron microscopy

## Abstract

Three-dimensional (3D) reconstruction of synaptic arrangement on a particular dendrite provides essential information regarding neuronal properties and neural microcircuits. Unconventional synapses are particularly good candidates for such steric attribution. In main and accessory olfactory bulbs (MOBs and AOBs), there are dendrodendritic reciprocal synapses (RSs) between excitatory projection neurons and inhibitory interneurons. Although the fine structure and configuration of these synapses have been investigated in MOB, their characteristics in AOB were unknown. In this study, we performed 3D AOB reconstruction using serial section transmission electron microscopy. We found numerous RSs on primary dendrites from glomeruli to mitral/tufted (MT) cell somas. These synapses formed between dendritic shafts of MT cells and large dendritic spines, or so-called gemmules, of granule (Gr) cells. This indicates that chemical signals received by a glomerulus are regulated in the primary dendrite of an MT cell before reaching its soma. In MOB, RSs are located on secondary dendrites and act as lateral and self-inhibiting following mitral cell depolarization. Our results indicate that AOB intrabulbar microcircuitry is quite different from that in the MOB.

## Introduction

Dendrodendritic reciprocal synapses (RSs) are unusual synaptic structures in the glomerular layer (GL) and external plexiform layer (EPL) that are constructed between excitatory projection neurons and inhibitory interneurons in olfactory bulb (Shepherd and Greer, 2004). In particular, dendrodendritic RSs between mitral/tufted (MT) cell and granule (Gr) cells in the EPL are important for odor discrimination and olfactory learning (Yokoi et al., 1995; Brennan and Keverne, 1997). In the main olfactory bulb (MOB), mitral cells have a single thick, primary dendrite that receives input from olfactory sensory neurons and forms glomeruli, with some long secondary dendrites that extend laterally and terminate in the EPL (Shepherd and Greer, 2004). The primary dendrite has a smooth surface and is partially myelinated (Burd, 1980), indicating that the primary dendrites of mitral cells in the MOB are specialized for the rapid transduction of olfactory information to cell somas. RSs in the EPL are primarily distributed on secondary dendrites of mitral cells, and glutamate released from the mitral cell dendritic shafts to Gr cell dendritic spines is followed by γ-aminobutyric acid (GABA) release back onto mitral cells or lateral inhibition of other mitral cells from Gr cells (Mori and Takagi, 1978; Shepherd and Greer, 2004).

The accessory olfactory bulb (AOB) has a laminar structure that is similar to the MOB, but recent studies have indicated real differences in cytoarchitecture, glomerular formation, and physiological and morphological properties of projection neurons between the 2 bulbs (Urban and Castro, 2005; Dulac and Wagner, 2006; Larriva-Sahd, 2008; Yonekura and Yokoi, 2008; Yokosuka, 2012). The most critical differences involve projection neuron morphology and connectivity. AOB MT cells have between 2 and 10 primary dendrites and receive olfactory input from multiple glomeruli (Del Punta et al., 2002; Wagner et al., 2006; Larriva-Sahd, 2008; Yonekura and Yokoi, 2008). Conversely, secondary dendrites are not conspicuous (Larriva-Sahd, 2008; Yonekura and Yokoi, 2008). RSs are also present in the GL and MT cell layer (MCL) in the AOB (Ichikawa et al., 1995; Matsuoka et al., 1997, 2004). The RSs in the MCL are thought to be a key contributor to pheromonal memory (Kaba and Keverne, 1988; Brennan and Keverne, 1989; Brennan, 1994; Matsuoka et al., 2004; Kaba and Huang, 2005). However, the localization, distribution, and density of RSs in MT and Gr dendrites are unclear. In this study, we performed 3D reconstruction of electron micrographs to clearly demonstrate that RSs in the MCL are formed between primary dendrites of MT cells and dendritic spines of Gr cells. Our result indicates that intrabulbar microcircuits are quite different between the MOB and AOB.

## Materials and methods

### Animals and slice preparations

All experiments were carried out according to the Guidelines for the Care and Use of Animals of the Tokyo Metropolitan Institute of Medical Science. Olfactory bulb slices were prepared from isoflurane-anesthetized Wistar rats between postnatal days 21 and 26. Sagittal sections of OB were prepared with a microslicer (VT1200S, Leica Microsystems), and AOB-containing slices were collected and allowed to recover for 1 h at 30°C in normal artificial cerebrospinal fluid (ACSF) containing (in mM) 124 NaCl, 2.5 KCl, 1.2 KH_2_PO_4_, 26 NaHCO_3_, 1.2 MgSO_4_, 2.5 CaCl_2_, and 25 D-glucose and were saturated with 95% O_2_–5% CO_2_ gas (Fujiwara-Tsukamoto et al., 2010).

For normal ultrastructural observation, we used 8–20 weeks old Wistar rats. After isoflurane anesthetization, rats were perfused and fixed with 2% paraformaldehyde and 2% glutaraldehyde in 0.1 M phosphate buffer (PB).

### Dye injections

Whole-cell patch-clamp methods were performed in single MT cells and/or Gr cells in the AOB under visual guidance using patch-clamp amplifiers (Axopatch 1D and/or Axopatch 200B, Axon Instruments) through glass patch electrodes filled with an internal solution containing (in mM) 140 K-gluconate, 2 NaCl, 1 MgCl_2_, 10 HEPES, 0.2 mM EGTA, 2 5′-ATP Na_2_, 0.5 GTP Na_2_, and 10 biocytin (pH 7.4). Biocytin was loaded into neurons by repetitive current injection (+300 pA, 500 ms at 1 Hz for 15 min), and the slices were further incubated in ACSF at 37°C for 30 min.

### Morphological observations

Dye-injected slices were fixed by 4% paraformaldehyde, 0.1% glutaraldehyde, and 15% saturated picric acid in 0.1 M PB for 1 h at room temperature. After washing, the slices were reacted with Fluoro Nanogold-streptavidin-Alexa Fluor 488 (1:400, Nanoprobes) in 10% Block Ace (Yukijirushi) and 0.1% Triton X-100 in 0.1 M PB. Alexa Fluor 488-labeled neurons were observed with a confocal microscope (LSM510, Zeiss).

### 3D reconstruction of electron micrographs

The fluorescently labeled slices were postfixed with 2% glutaraldehyde in 0.1 M PB for 10 min. After several rinses, the gold particles were enhanced using a Silver Enhancing Kit (BB International) for 12 min. For electron microscopy, the slices were postfixed in 2% glutaraldehyde in 0.1 M PB for 10 min, incubated in 1% osmium tetroxide in 0.1 M PB for 1 h, and then dehydrated in a graded series of ethanol followed by propylene oxide. Each slice was embedded between 2 flat Acrafilms (Nisshin EM) in epoxy resin (Quetol 812, Nissin EM). The embedded slices were viewed under a light microscope, and the target regions containing labeled primary dendrites were trimmed. Serial semi-thin sections (4-μm thickness) were cut from the region, and appropriate sections were re-embedded. A total of 50–80 ultrathin serial sections (silver/gray interference color) were cut from the region and placed in formvar-coated, single-slot grids. After staining with uranyl acetate and lead citrate, ultrathin sections were observed under a transmission electron microscope (H-7650, HITACHI). Images of labeled dendrites were digitally recorded at a magnification of 3000. Electron micrographs were consecutively aligned using Photoshop (Adobe) to identify the synaptic types on labeled dendrites. Synaptic directions were determined by their morphology on electron micrographs: location of postsynaptic density (PSD) and synaptic vesicles. The serial images were exported to Reconstruct free-software (http://synapses.bu.edu/) to generate 3D reconstruction images.

## Results

### Synaptic properties of MT cell primary dendrites in the AOB

**Figure 1:**
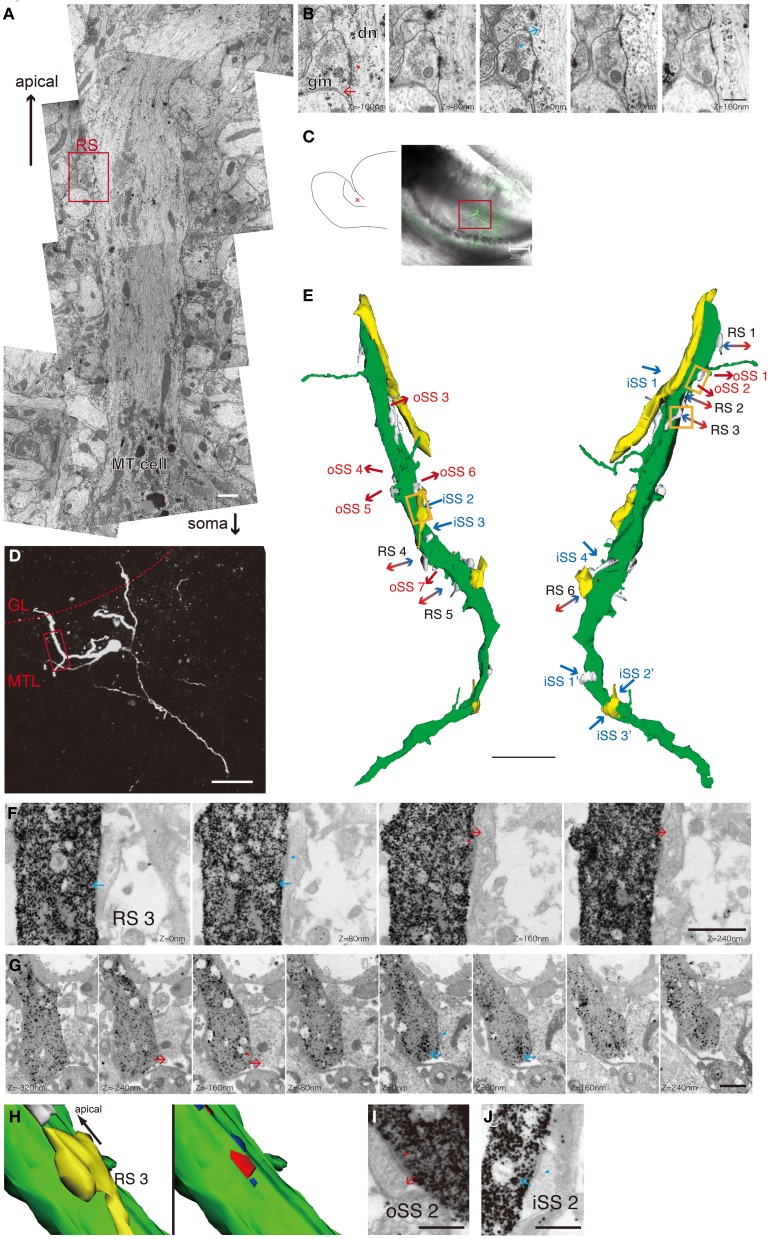
**Ultrastructual morphology and 3D images of MT cell primary dendrites in the AOB. (A)** Basic morphology of the MT cell apical dendrite *in vivo*. The sample was fixed in general fixing solution for electron microscopic observation. We can recognize the dendrite was MT cell's without labeling if we observed the proximal region of the neuron. Scale bar = 1 μm. **(B)** A serial micrograph of an RS in the red square in **A** dn, an apical dendrite of a MT cell; gm, a gemmule of a Gr cell. Arrows indicate synaptic transmission direction. Arrowheads indicate docked synaptic vesicles. Scale bar = 500 nm. **(C)** A schematic drawing of a rat AOB slice preparation (left panel) and the position of a biocytin-injected MT cell (red cross, left panel), and a merged image of differential interference and fluorescence of the slice reacted with avidin-FITC-gold (right panel). The red box indicates the area shown in **D**. Scale bar = 200 μm. **(D)** A confocal fluorescent image of the injected cell. The red dotted line indicates the border between the GL and MTL. The red box indicates the observed area by electron microscopy corresponding to the 3D image in **E**. Scale bar = 50 μm. **(E)** A reconstructed 3D image of the primary dendrite in **D** by serial electron micrography. The green object is the injected dendrite. The yellows and whites are the structures of other neurons synapsing with the MT cell. Arrows indicate synaptic transmission direction. oSSs (all excitatory synapses) are labeled in red, iSSs (mainly inhibitory synapses) are labeled in blue, and bidirectional arrows indicate RSs. Orange boxes indicate the area of **F** and **H–J**. Scale bar = 5 μm. **(F)** The serial electron micrographs of RS3 in **E**. Serial electron micrographs indicate steric composition of a labeled dendrite by silver-enhanced gold particles. Arrows indicate synaptic transmission direction. Scale bar = 1 μm. **(G)** The RS on another labeled MT cell. These micrographs more clearly identified intracellular structures, such as synaptic vesicles. Arrows indicate synaptic transmission direction. Arrowheads indicate synaptic vesicles. Scale bar = 500 nm. **(H)** The 3D image of RS3 reconstructed by serial picture in **F**. The right panel indicates the locations and sizes of synaptic sites (red, PSD of paired cell; blue, PSD of the labeled cell). Yellow indicates a synaptic pair structure. This RS contains 1 output and 2 input synapses. Scale bar = 1 μm. **(I** and **J)** Sample images of an oSS (oSS2) and an iSS (iSS2). Scale bars = 500 nm.

In the AOB, the designation “MT cell” is a general term for projection neurons in MTL (Takami and Graziadei, 1991). MT cells have the largest somas in the AOB, but these are smaller than the somas of typical MOB mitral cells. MT cell bodies are distributed within the MTL. AOB MT cells have various morphological properties (Takami and Graziadei, 1991; Yonekura and Yokoi, 2008); however, it is not clear whether cell morphologies are related to synaptic connections and physiological characteristics. First, we confirmed RS 3D morphologies using adult rat AOBs by general method for electron microscopy. We observed thick dendrites protruding from MT cell somas and extending to the apical layer in serial thin sections (Figure 1A). We could easily identify RSs on the dendritic shafts of MT cells and confirmed the ultrastructural properties of RSs. In excitatory synaptic sites of RSs, docked synaptic vesicles were seen on MT dendrites, and electron-dense PSD were recognized in gemmules of Gr cell (Figure 1B, *Z* = 160−μm section). Another section (*Z* = 0 μm, same as Figure 1A) showed inhibitory synaptic sites of the same RS. It contained non-uniform synaptic vesicles and docked vesicles in gemmules and thin PSDs in MT dendrites. This method provided us an abundance of ultrastructual information, but it was not possible to enough match the Z-axis through serial sections. Therefore, we could not reconstruct long dendrites and determine which ones really extended into glomeruli.

Next, we identified single-cell morphologies using the patch-clamp method to inject biocytin and identify primary dendrites that extended into the GL, where we observed their ultrastructure. We visualized 27 MT cells from 41 acute slices using FITC and gold particle-conjugated avidin, and we selected cells with complete primary dendrites extending into the GL by confocal imaging. A sample cell is shown in Figures 1C,D. Primary dendritic regions in the MT cell layer were identified and reconstructed using serial-section electron microscopy. We obtained 4 reconstructed dendrites more than 20 μm long.

**Figure 2:**
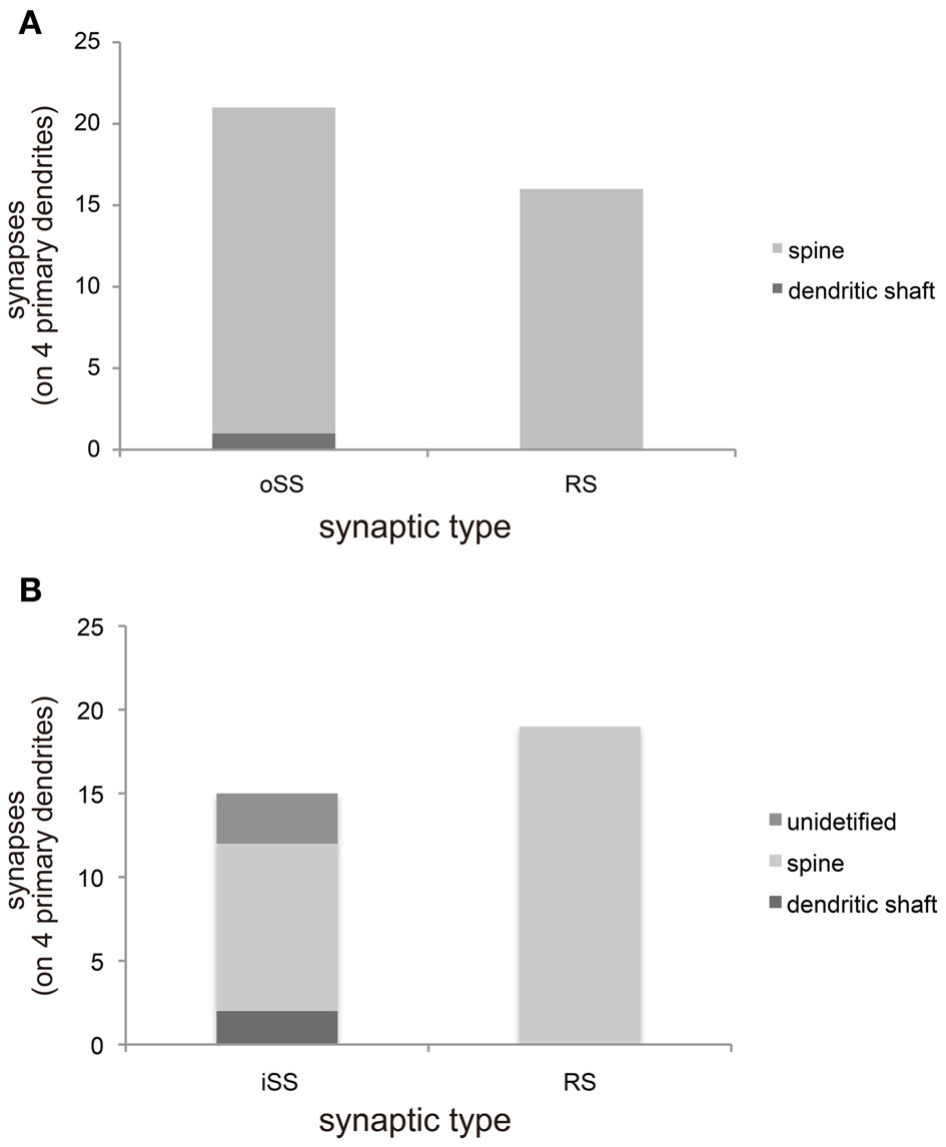
**Morphological characterizations of synaptic sites. (A)** The constitution of output synapses from MT cell primary dendrites to other neurons from 4 primary dendrites. These synapses categorized by synaptic type [single synapse (oSS) or reciprocal synapse (RS)] and postsynaptic architecture of paired neurons. Most synapses formed with dendritic spines. **(B)** The construction of input synapses from other neurons to primary dendrites of MT cells. These synapses were categorized by synaptic type (iSS or RS) and presynaptic architecture of paired neurons. All RSs formed with dendritic spines; however, presynaptic structures of iSSs were not uniform.

We categorized synaptic type by synaptic direction into 3 types: dendrodendritic RS (Figures 1F and G), output single synapse (oSS, Figure 1I), and input single synapse (iSS, Figure 1J). All oSSs in MT cells are glutamatergic, but iSSs are not uniform and might contain inputs from other interneurons and afferent fibers. Synaptic densities were 1.5 ± 0.5 (RS), 2.1 ± 0.6 (oSS), and 1.2 ± 0.5 (iSS) per 10 μm (mean ± S.D., *n* = 4). This indicates that approximately 30% of synapses were RSs, and others were single synapses. It is known that primary dendrites of MT cells are aspiny. Ultrastructural 3D reconstruction revealed some protrusions, but these structures did not form synapses (Figure 1E). All synapses on primary dendrites in the MT cell layer were found directly on dendritic shafts (Figure 1E). Presynaptic sites on MT cells mainly transmitted to dendritic spines of other cells (Figure 2A). All RSs were between MT cell dendritic shafts and perhaps Gr cell dendritic spines (Figures 1F,G and Figures 2A,B). Single synaptic inputs were received from various structures (Figure 2B). Some RSs were comprised of 1 excitatory synapse and 2 inhibitory synapses (Figure 1H).

### Gr cell dendritic morphology

The majority of AOB GABAergic interneurons are Gr cells (Larriva-Sahd, 2008). Gr cell bodies are located in the GRL and are tightly adjoined. Interneurons subtypes in the GRL, except Gr cells, were also observed (Nakajima et al., 1996; Larriva-Sahd, 2008). We focused on typical Gr cells, which are assumed to form RSs with MT cells. Typical Gr cells are defined as (1) having bipolar dendrites with at least 1 apical dendrite extending into the MTL, (2) having a small round soma (less than 10 μm), (3) having large spines (so-called gemmules) on apical dendrites in MTL, and (4) lacking an axon (Larriva-Sahd, 2008; Figure 18C-c).

**Figure 3:**
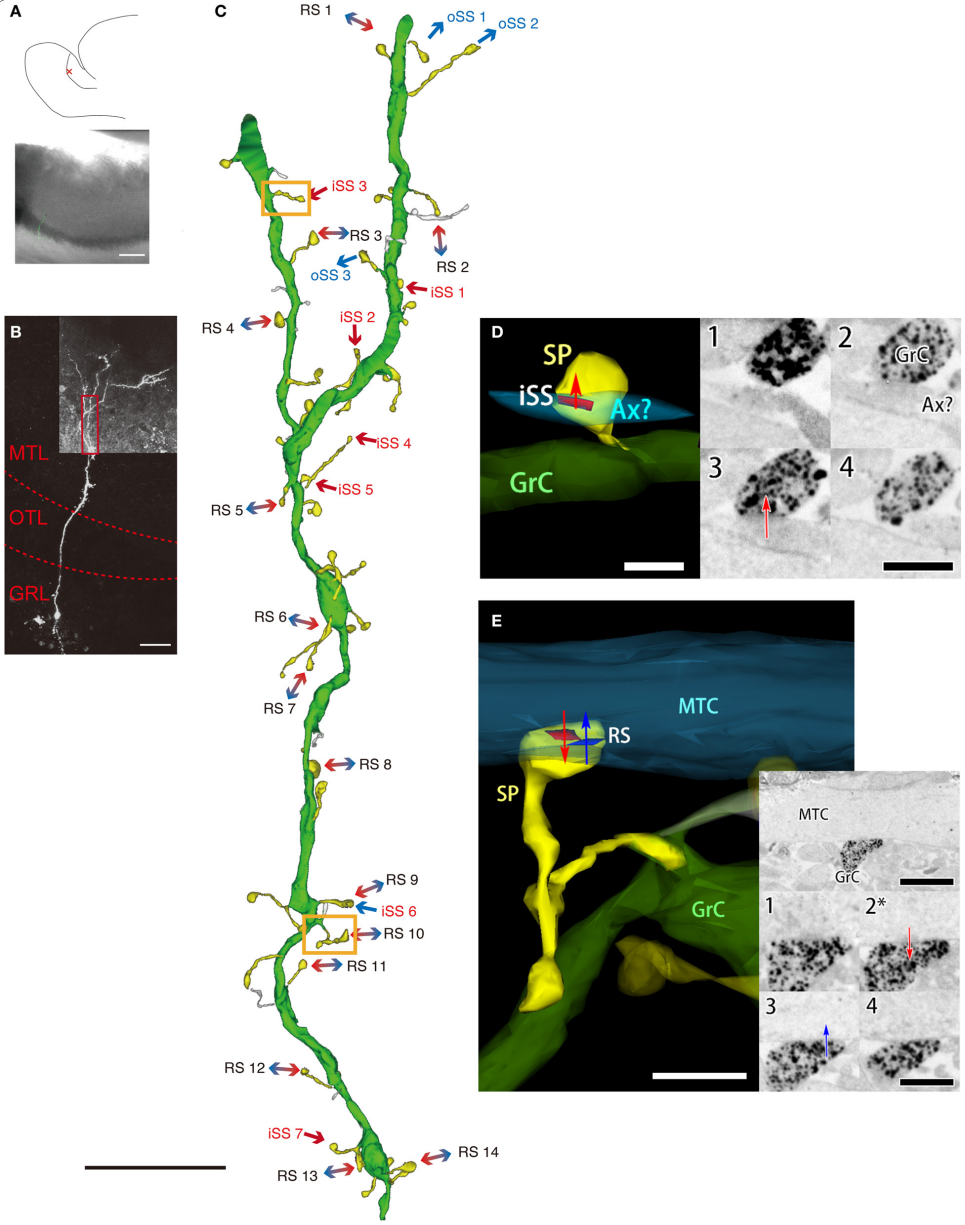
**A 3D image of an AOB Gr cell. (A)** A drawing of a prepared rat AOB slice (upper panel) and the position of a biocytin-injected Gr cell (red cross, upper panel), and a joint image of differential interference and fluorescence of the slice reacted with avidin-FITC-gold (lower panel). Scale bar = 200 μm. **(B)** A confocal fluorescent image of the injected cell. The red dotted line indicates the border between the MTL, the olfactory tract layer (OTL), and the GRL. The red box indicates the area observed with electron microscopy corresponding to the 3D image of **C**. Scale bar = 50 μm. **(C)** 3D reconstruction of the apical dendrite in **B** by serial electron micrograph. The green is the injected dendrite, and the yellow structures indicate the neuron's dendritic spines. The white structures are filopodia-like structures. Arrows indicate synaptic transmission direction. iSSs (mainly excitatory synapses) are labeled in red, oSSs (all inhibitory synapses) are labeled in blue, and bidirectional arrows indicate RSs. Orange boxes indicate the areas shown in **D,E**. Scale bar = 10 μm. **(D** and **E)** An example of 3D morphology of the spine containing the iSS (iSS3 in **C**) and the gemmule containing the RS (RS10 in **C**). The 3D image (left panels) was reconstructed with the serial electron micrograph of a labeled spine by silver-enhances gold particles (right panels). Numbers on the right panels indicate ultrathin section sequence. The asterisk indicates the same section of the low-magnification panel (upper). Arrows indicate synaptic transmission direction. Red and blue areas indicate the locations and sizes of synaptic sites (red, PSD of the labeled cell; blue, PSD of a paired MT cell). Ax: axon-like structure. White scale bar = 500 nm (in **D**) and 1 μm (in **E**). Black scale bar = 500 nm (in **D**), 1 μm (low-magnification photo in **E**), and 500 nm (high-magnification photos in **E**).

To reconfirm RS synaptic structure, we visualized Gr cells with the same method used for MT cells. We attempted 36 slices; however, only 5 typical Gr cells were completely visualized. These cells were embedded and sectioned for electron microscopy, but we were only able to construct one 3D image of a typical Gr cell using serial electron micrographs (Figure 3). We observed Gr cell dendrites in the MTL (Figure 3B, red box). In this region, dendritic spine density was 4.1 spines per 10 μm; however, 23 out of 49 spines were not explicitly recognized to have synaptic structure (Figure 3C). All synapses, including only presynaptic sites of the labeled Gr cell, were present on dendritic spines. On the other hand, 14 filopodia-like structures that did not have spine heads larger than spine neck diameter, did not form synapses (Figure 3C). Most detected synapses were RSs (13 synapses), but iSSs (8 synapses) and oSSs (6 synapses) were also observed (Figure 3C). In Gr cell, oSSs were GABAergic, but the neurotransmitters in iSSs were not distinctly identified. The spine heads containing RSs (0.244 ± 0.063 μm^3^, mean ± S.D.; *n* = 14; Figure 3E) were larger than that of iSS spines (0.095 ± 0.028 μm^3^, mean ± S.D.; *n* = 8; Figure 3D). To reinforce this difference in volume between RSs and single synapses, we calculated spine volumes of that on MT cells from MT cell-reconstruction images. We chose the calculated spines that contacted labeled MT cells and had fully reconstructed 3D images. The average RS-containing spine volume on labeled MT cells was 0.199 ± 0.166 μm^3^ (mean ± S.D., *n* = 6), and the average single-synapse spine volume was 0.116 ± 0.061 μm^3^ (mean ± S.D., *n* = 12). It is predicted that these data from MT cell reconstructions also included non-Gr cell spines. Nevertheless, the results indicated that RS spines are approximately twice as large as single-synapse spines. It was concluded that most Gr cell gemmule structures are likely to contain RSs.

## Discussion

Morphological differences between AOB and MOB projection neurons have been reported (Urban and Castro, 2005; Dulac and Wagner, 2006; Larriva-Sahd, 2008; Yonekura and Yokoi, 2008), but synaptological differences have not been described. In this study, we were able to quantify synapses on the primary dendrites of AOB MT cells. Including dendritic RSs, the average density of output synapses was 3.6 in 10 μm, and that of input synapses was 2.7 in 10 μm.

Approximately 30% of all synapses were RSs. In 1 ultrathin section, RSs accounted for less than 10% of all synapses in the MTL. This suggests that 3D reconstruction provides important data and is useful for assessing AOB functions. We found that MT cell primary dendrites received some inputs, perhaps inhibitory, from sources other than RSs. In the MTL, inhibitory feedback input from the bed nucleus of stria terminalis has been reported (Fan and Luo, 2009), and some inputs were from those afferent axon terminals. Another possibility is input from small interneurons, such as dwarf cells in the MTL (Larriva-Sahd, 2008). It indicates that MT cell primary dendrites are influenced by other inhibitory neurons.

We were only able to reconstruct 1 complete 3D image of a Gr cell. Although more data is required to perform a quantitative analysis, we did observe that some spines did not form synapses. However, it is not clear whether the data reflect *in vivo* conditions. It is possible that some synapses were disrupted during acute slice preparation and dye injection. Another possibility is that the sampled rat was not fully developed at postnatal day 26. Thus, additional work is necessary to confirm our findings.

Our results verify that MT cell primary dendrites receive dendrodendritic input from Gr cells and provide output to Gr cells. This structure supposes that MT cells receive feedback inhibition from Gr cells before excitatory input reaches cell somas. It suggests that RSs are specialized to regulate chemical signals, such as pheromones, from glomeruli to cell somas, and regulate MT cell firing rates. This hypothesis is supported by a previous report that glomerular calcium (Ca^2+^) influx does not synchronize with MT cell somas (Urban and Castro, 2005). Gr cells receive numerous inputs in the granule cell layer (GCL) from the piriform cortex, posteromedial cortical amygdala, anterodorsal medial amygdala, ventral subiculum (Mohedano-Moriano et al., 2012), and locus coeruleus in the brainstem (McLean and Shipley, 1991). These inputs might modulate synaptic transmission frequency and intensity in RSs, and Gr cells might regulate the MT cell-firing ratio. A recent study suggests that AOB MT cells exhibit more prolonged firing induced by strong stimulation than those in the MOB because of these intrinsic properties (Shpak et al., 2012). Therefore, if the environmental situation is not suitable for sending pheromonal signals from vomeronasal neurons to higher brain areas, it is necessary to block pheromonal transduction. In this scenario, Gr cells inhibit MT cell excitation before transducing pheromonal input to cell somas via RSs.

**Figure 4:**
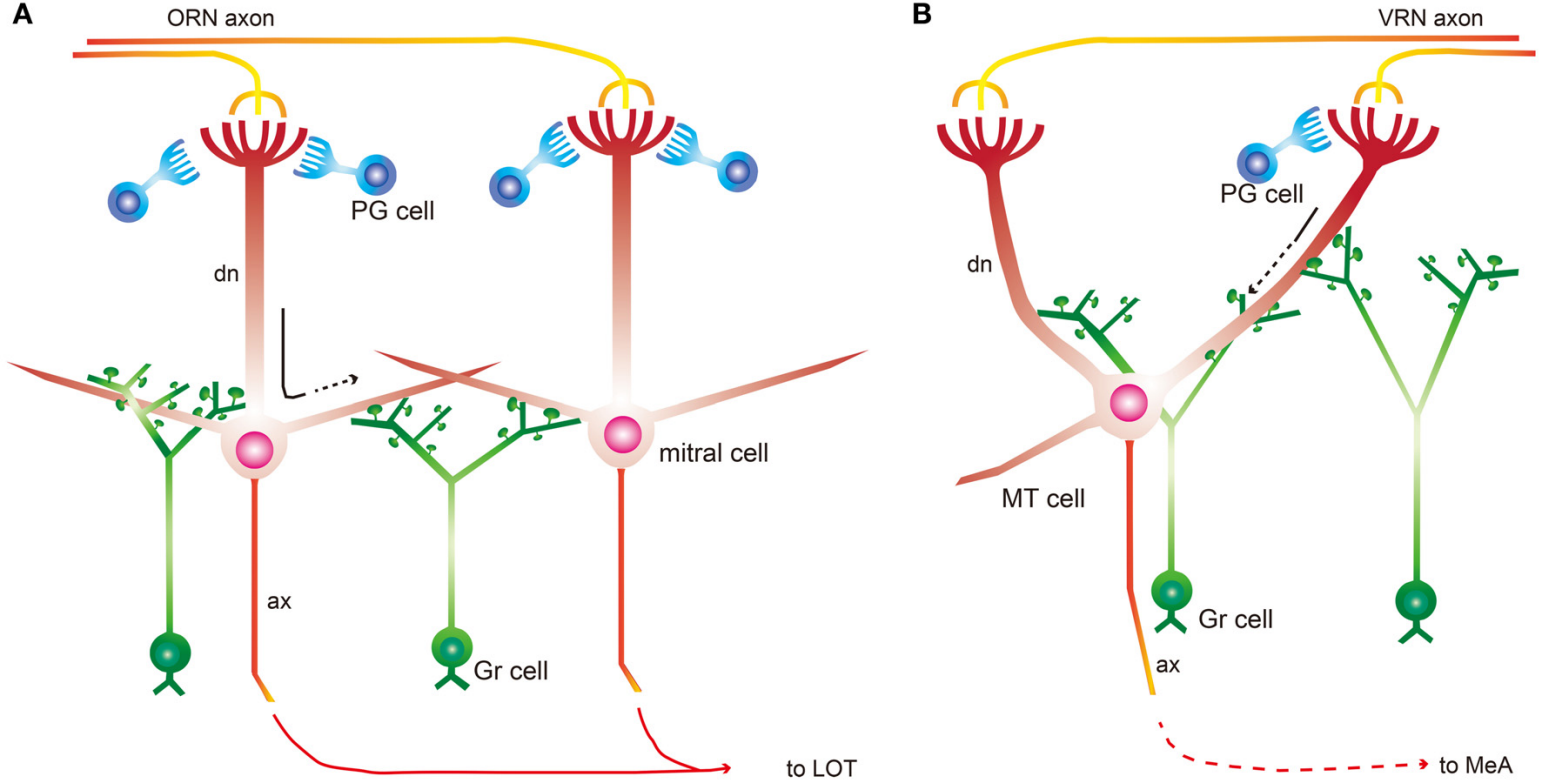
**Schematic summarizing RS location and function. (A)** A simplified model of the MOB intrabulbar circuit. Mitral cells receive odor input from olfactory receptor neurons (ORN) and then send olfactory information to the olfactory cortex through the lateral olfactory tract (LOT). Mitral cells receive inhibitory input from mainly periglomerular (PG) and Gr cells. Gr cell spines form RSs with mitral cells primarily on secondary dendrites. This dendrodendritic signaling is driven by mitral cell soma depolarization. RSs act through lateral inhibition and self inhibition after depolarization for effective discrimination. Black arrow indicates the dendritic conduction of excitatory odor cues. **(B)** A simplified model of the AOB intrabulbar circuit. MT cells receive chemical information from vomeronasal receptor neurons (VRN) through several glomeruli and then send the information to the medial amygdala (MeA). PG cells in the AOB are not as numerous as in the MOB. MT cells in the AOB form RSs with Gr cells on primary dendrites. RSs in the AOB act as gate keeper before relaying the chemical cue to cell somas. Black arrow indicates the dendritic conduction of excitatory chemical signals, and dotted line indicates the attenuation of excitation.

We provide evidence for our hypothesis regarding RS profile differences between the MOB and AOB in Figure 4. In the MOB, RSs function as a rapid suppressor of intra- and inter-neuronal excitation for integrated discrimination and are primarily located on secondary dendrites of mitral and tufted cells. In contrast, AOB RSs gate signal sending. Gr cell morphology is quite similar in both bulbs, but gene expression (e.g., Ca^2+^-binding proteins and neurotransmitter receptors) are different (Kaba et al., 1994; Kosaka et al., 1994a,b). These molecules might mediate physiological differences in Gr cells between the 2 bulbs. However, many basic Gr cell properties might be same. It is reported that transmission in MOB Gr cell gemmules is triggered by local Ca^2+^ influx (Abraham et al., 2010), suggesting that Ca^2+^ influx is a key factor underlying RS function. It is not clear whether this system is also in the AOB, but the existence of the same local Ca^2+^ mechanism is supported by the fact that the AOB contains similar gemmule structures.

In this study, we elucidated a portion of 3D AOB synaptology. Our results show the utility of the 3D reconstruction technique for understanding brain microcircuits because it allows us to decipher the neurological and physiological features of specific morphological characteristics.

### Conflict of interest statement

The authors declare that the research was conducted in the absence of any commercial or financial relationships that could be construed as a potential conflict of interest.
